# Varroa and Colony Losses: Practical Advice for Hobbyist Beekeepers

**DOI:** 10.3390/insects17080870

**Published:** 2026-08-21

**Authors:** Philip Stahlmann-Brown, Ken Brown, Richard Hall, Frank Lindsay, Hayley Pragert, Michelle Taylor

**Affiliations:** 1 Motu Economic and Public Policy Research, Wellington 6011, New Zealand; 2 Land and Water Solutions, Wellington 5013, New Zealand; 3 Ministry of Bees, Auckland 0882, New Zealand; 4 Auckland Beekeepers Club, Auckland 1025, New Zealand; 5 Ministry for Primary Industries, Upper Hutt 5018, New Zealand; 6 Lindsay’s Apiaries, Wellington 6037, New Zealand; 7 Southern North Island Beekeepers Group, Newbury 4478, New Zealand; 8 Ministry for Primary Industries, Auckland 2022, New Zealand; 9 New Zealand Institute for Bioeconomy Science, Hamilton 3214, New Zealand

**Keywords:** *Varroa destructor*, *Apis mellifera*, integrated pest management, varroa management, New Zealand Colony Loss Survey, COLOSS

## Abstract

*Varroa destructor* is the leading cause of honey bee colony losses globally and is increasingly implicated in winter colony mortality in New Zealand. This study used data from the New Zealand Colony Loss Survey to evaluate the relationship between specific varroa management practices and over-winter colony losses among 5203 hobbyist beekeepers surveyed between 2023 and 2025. We find that beekeepers who did not treat for varroa experienced loss rates of 49.4% compared with 30.7% among beekeepers who treated. Conditional on treatment, colony losses declined as the diversity of products used increased and as treatment applications were spread throughout the year. For example, beekeepers using four or more products experienced losses of 21.2%, while those treating in three or more seasons of the year experienced losses of 24.8%. Multiple autumn treatments were also associated with lower loss rates. No statistically significant differences were observed among major treatment types when hobbyist beekeepers used a single treatment type in a single season of the year. While apiary size is negatively associated with loss rates, beekeeper experience is not. The results demonstrate that treating for varroa, product rotation, treatment throughout the year, and intensified autumn control are associated with substantially improved over-winter colony survival among hobbyist beekeepers.

## Simple Summary

Varroa mites are the biggest threat to honey bees globally. These parasites weaken bees, spread viruses, and lead to colony death if not controlled. This study uses data from more than 5200 New Zealand hobbyist beekeepers (among whom colony loss rates are very high) collected between 2023 and 2025 to identify practical ways to manage varroa and reduce colony losses. Treating colonies for varroa is the single most important action beekeepers can take to improve colony survival. Hobbyist beekeepers who did not treat for varroa lost about half their colonies over winter, while those who treated lost about 31%. Survival rates were also higher among beekeepers who used more than one type of treatment, treated throughout the year, and applied more than one treatment during autumn. Each of these practices was linked to lower colony losses. Finally, while apiary size is correlated with loss rates, beekeeper experience is not. The empirical evidence is strong: treat for varroa, rotate products, and maintain control throughout the year to improve colony survival.

## 1. Introduction

The ectoparasitic mite *Varroa destructor* (hereafter, ‘varroa’) is among the most serious threats to honey bees globally. When a varroa mite feeds on a bee, it causes substantial damage to the fat body tissue, an organ that is essential for immune function, energy storage, and detoxification [1]. This parasitism weakens individual bees and assists viral replication, compounding impacts on colony health [2]. Varroa is also a vector for many viruses, particularly *Iflavirus aladeformis* (deformed wing virus, DWV), as it enables amplification and transmits the viruses within and among bees [3]. Uncontrolled varroa infestations typically lead to colony mortality within a few years, often much sooner [4,5], particularly in places where brood-breaks are uncommon.

The impact of varroa is determined by its population dynamics, which follow the seasonal brood cycles of honey bee colonies. Because mites exclusively reproduce within capped brood cells [6,7], mite populations within a colony can quickly increase during the spring and summer when there is a lot of brood. As brood declines in late summer and autumn, varroa shift onto adult bees, increasing the proportion of phoretic mites and measured infestation levels [8,9]. Varroa reinvasion from neighbouring apiaries after treatment or late in the season may also intensify due to robbing behaviour [8,10,11]. Together, these effects produce high parasite burdens as colonies begin producing long-lived winter bees; varroa-associated DWV reduces winter-bee lifespan and contributes to over-winter failure [12]. Late-summer and autumn varroa infestation levels are among the strongest predictors of over-winter loss as virus loads peak and colony homeostasis is disrupted [13,14,15].

It follows that winter survival of a honey bee colony can be fostered by good varroa management, which depends entirely on the beekeeper. The scientific consensus is that effective mite control requires routine monitoring, the use of defined treatment thresholds, rotation among treatment types, and responsive treatment timing [16,17,18].

Varroa treatments include synthetic miticides and organic acids as well as mechanical methods. Amidines such as amitraz affect neural transmission by increasing the octopamine receptors in cells [19]. Pyrethroids such as tau-fluvalinate and flumethrin cause cell dysfunction by changing sodium channels [20]. Thymol and other essential oils disrupt neural functioning [21]. Formic acid reduces oxygen availability to varroa mites [20], and oxalic acid inhibits dietary function by binding to minerals such as iron, calcium, and sodium [22]. Mechanical methods include queen caging and drone brood removal.

Despite global awareness about the harm caused by varroa and the scientific evidence on effective management (e.g., Google Scholar currently returns more than 27,000 research articles on the search term “*Varroa destructor*” and more than 1500 research articles for the terms “varroa treatment” and “varroa treatments”), beekeeper management practices still remain varied. For example, results from the annual New Zealand Colony Loss Survey (NZCLS) show that beekeepers differ in whether they monitor for mite infestation levels, whether they apply treatments, when they treat, how many times they treat, and which products they use [23]. All such differences may impact colony survival.

Varroa losses are especially acute amongst hobbyist beekeepers in New Zealand. For example, in 2025, the winter loss rate for New Zealand beekeepers with fewer than 50 colonies (hereafter “hobbyist beekeepers” or “hobbyists”) was 33.4%. Among beekeepers with more than 500 colonies (hereafter, “commercial beekeepers”), the corresponding loss rate was 13.0%, equating to a 2.5-fold difference [24]. Indeed, over-winter loss rates among these hobbyist beekeepers have exceeded loss rates among commercial beekeepers in each of the last seven surveys (Table 1). This difference is not limited to New Zealand: for example, in the US, hobbyist beekeepers lost 45.5% of their colonies over winter 2016–2017 compared to 25.6% for commercial beekeepers [25,26]. Brodschneider et al. [27] report higher loss rates among small operators (defined as 1–50 colonies) than larger operators in Austria, Denmark, Estonia, Finland, Germany, Ireland, Italy, Latvia, Norway, Poland, Spain, Sweden, Switzerland, Türkiye, and Ukraine, but not in Algeria, France, Israel, or Slovenia (in other countries that were surveyed, too few beekeepers with more than 50 colonies were included to analyse results meaningfully). Indeed, operation size is frequently identified as an important factor in loss rates [27,28].

Evidence based on survey data can improve colony health and reduce honey bee mortality rates among hobbyist beekeepers [29]. Using three years of annual NZCLS data, our analysis quantifies the benefits of varroa control for hobbyist beekeepers and provides practical, evidence-based guidance. We focus on how treatment affects survival rates, the benefits of using diverse products to treat for varroa, whether treating for varroa in multiple seasons (e.g., spring and autumn, as opposed to one of them) improves survival, whether multiple autumn treatments are advantageous, the relative effectiveness of products to treat varroa, and the effects of beekeeper experience on colony loss rates.

## 2. Materials and Methods

### 2.1. New Zealand Colony Loss Survey

The NZCLS has been conducted annually since 2015 [30]. The questionnaire is based on the standardised international COLOSS survey [31] but has been adapted to the New Zealand context. For example, the NZCLS includes detailed information on colony predation by wasps and losses due to theft and vandalism, both of which are important local concerns. Similarly, questions focusing on varroa management emphasise treatments that are registered in New Zealand, and questions focusing on pollination services emphasise specific crops such as kiwifruit and avocados.

Under the Biosecurity Act 1993, all New Zealand beekeepers are required to register their hives. In 2025, 98.7% of beekeeper registrations included email addresses, consistent with New Zealand’s Internet penetration exceeding 96% [32]. The survey is administered online via the Qualtrics survey research platform. Beekeepers are invited to complete the survey via personalised emails, and reminders are sent at 15-day intervals. In addition, the survey was promoted through magazines such as the Apiarist’s Advocate and the New Zealand BeeKeeper, online discussion groups, and beekeeping clubs. The survey is open from 1 September through to the end of November each year.

The NZCLS employs branching to minimise the time commitment, and the questionnaire is optimised for completion on mobile devices as well as computers. Response rates are consistently world-leading, with between 25% and 50% of all beekeepers and between 25% and 50% of all registered bee hives represented in the survey, depending on the year [24]. Table 2 reports the number of beekeepers and the number of colonies they manage for the three survey years used in this analysis: 2023, 2024, and 2025.

### 2.2. Varroa Treatment in New Zealand

Nearly all New Zealand beekeepers treat varroa with synthetic miticides and/or organic acids [24]. Table 3 describes the products that are commonly used to treat varroa in New Zealand.

### 2.3. Empirical Strategy

Country-level reporting in colony loss surveys is based on aggregated data, either recording the average loss rate across beekeepers (calculated as the mean of individual beekeepers’ loss rates, with each respondent’s losses expressed as a proportion of the number of colonies alive at the beginning of winter) or the overall loss rate (calculated as the total number of colonies lost across all respondents divided by the total number of colonies alive at the beginning of winter). See, for example, [28,34,35,36].

In contrast, the present study focuses on the individual loss rate of hobbyist beekeepers (defined as managing 1–49 colonies), i.e.,

(1) $$R_{i}=\frac{L_{i}}{C_{i}}$$

where $R_{i}$ is the individual loss rate for hobbyist beekeeper i, L is the number of colonies they lost over winter, and C is the number of colonies they managed at the beginning of winter. Due to lower average levels of experience, lower dependence on beekeeping as a source of income, and different management priorities [37], hobbyist beekeepers consistently report higher individual loss rates and different management practices than sideline and commercial beekeepers (e.g., [27,28,37,38]).

More New Zealand beekeepers attribute over-winter losses to varroa than to any other cause [39]. Indeed, the study in [24] estimates that 6.4% of healthy hives entering winter 2023, 4.6% of healthy hives entering winter 2024, and 7.0% of healthy hives entering winter 2025 died as a result of varroa and related complications. Losses attributed to varroa exceeded those attributed to all other causes combined in 2023 and 2025; in 2024, they exceeded the combined losses attributed to queen problems, wasps, and starvation. In 2024 and 2025, estimated varroa losses were higher among hobbyists than in any other size class; in 2023, varroa losses among beekeepers managing 50–499 colonies exceeded those of hobbyists [24,40,41].

Our empirical approach focuses on how varroa management affects individual loss rates amongst hobbyist beekeepers. Because loss rates are bounded, i.e. $R_{i}\in \left[0,1\right]$, Ordinary Least Squares regression can produce fitted values outside the unit interval, and log-odds transformations are undefined at $R_{i}=0$ and $R_{i}=1$. Therefore, we estimate a series of fractional response probit models in which the conditional mean is specified as

(2) $$E\left(R_{i}\;|\;M_{i},X_{i}\right)=\Phi \left(\beta_{0}+\beta_{1}M_{i}+\beta_{2}^{\prime }X_{i}\right)$$

where $\Phi \left(\cdot \right)$ is the standard normal CDF, M indexes specific interventions for managing varroa employed, and *X* is a vector of controls that includes the beekeeper’s experience, apiary size, the survey year, the beekeeper’s home region, and year × region interactions to account for temporal and spatial patterns in loss rates. Specific varroa management interventions include whether beekeepers controlled varroa at any point between the previous spring and the current winter; the number of different products used (conditional on treating varroa); the number of seasons (i.e., spring, honey flow, autumn, winter) in which treatments were applied (conditional on treating varroa); whether multiple autumn treatments were used (conditional on treating varroa in autumn); and the specific treatment applied (conditional on whether a single varroa treatment was used during a single season).

Parameters are estimated using quasi-maximum likelihood, maximising $\sum_{i=1}^{N}l_{i}\left(\beta \right)$, where the contribution of the ith beekeeper to the quasi-log-likelihood is as follows:

(3) $$l_{i}\left(\beta \right)=R_{i}\ln\;[\Phi \left(\beta_{0}+\beta_{1}M_{i}+\beta_{2}^{\prime }X_{i}\right)]+\left(1-R_{i}\right)\ln\;[1-\Phi \left(\beta_{0}+\beta_{1}M_{i}+\beta_{2}^{\prime }X_{i}\right)]$$

$\hat{\beta }$ is a consistent estimator, provided that the conditional mean is correctly specified. Since $\beta_{1}$ is not itself a marginal effect, we report the average partial effect, ${\hat{\beta }}_{1}\cdot N^{-1}\sum_{i}\phi \left({\hat{\beta }}_{0}+{\hat{\beta }}_{1}M_{i}+{\hat{\beta }}_{2}^{\prime }X_{i}\right)$, together with heteroskedasticity-robust standard errors.

Varroa monitoring is a critical tool in colony management because it helps pinpoint the optimal timing for treatment (e.g., [9]). Specifically, poorly timed treatment may be ineffective and/or reduce colony health [42], and thus treatment is ideally undertaken when mite loads reach a prescribed “economic threshold” [16,43], preventing the mites from reaching a level that causes economic injury [18,44]. Indeed, treatment undertaken with high mite loads may not reduce mite numbers sufficiently to prevent colony losses [14,45]. That is to say, simultaneity bias may arise when estimating Equation (2). If beekeepers treat reactively after monitoring shows mite loads beyond the economic threshold, then estimates of the effectiveness of treatment may be downward-biased. Therefore, to eliminate this potential source of simultaneity bias, we also estimate Equation (2) with a restricted sample consisting of beekeepers who undertook no varroa monitoring. The NZCLS records the following activities as monitoring: alcohol or detergent wash, sugar shake/roll, CO_2_, sending samples to a lab, sticky board (or other collection tray below the hive), and uncapping drone brood; visual inspection of adult bees is not considered varroa monitoring.

### 2.4. Summary Statistics

Table 4 shows summary statistics for individual loss rates, management interventions, and control variables for all hobbyist beekeepers that contributed to the NZCLS. The overall sample comprises 5203 hobbyist beekeepers, including 2053 in 2023, 1907 in 2024, and 1243 in 2025. On average, hobbyist beekeepers lost 26.3% of their colonies over winter 2023, 33.8% of their colonies over winter 2024, and 34.2% of their colonies over winter 2025.

More than 98% of hobbyist beekeepers treated varroa in each of the three years. Most commonly, they used two different products in two different seasons to treat for varroa, although 5.6% of beekeepers treated with four or more products and 29.2% treated in three or more seasons, e.g., spring, autumn, and winter. Among beekeepers who treated in autumn, 48.1% applied multiple autumn treatments.

Across the three survey years, there were 826 cases in which hobbyist beekeepers applied only one treatment type in only one season. Among this subsample, amitraz (marketed in New Zealand as Apitraz^®^ and Apivar^®^) was applied by 39.0%, flumethrin (marketed as Bayvarol^®^) was applied by 32.9%, and oxalic acid (vaporisation, dribbling, or glycerine strips) was applied by 11.0%. Other treatments including formic acid and thymol were applied by 17.1% of beekeepers. However, the percentage of beekeepers using each treatment evolved between 2023 and 2025, with amitraz and flumethrin use declining and oxalic acid and other treatments becoming more common.

For the control variables, 47% of hobbyist beekeepers in the study reported having more than five years of beekeeping experience. The average apiary size is 3.81 colonies. Nearly two-thirds of hobbyist beekeepers live in the North Island.

Varroa monitoring was conducted by 77.7% of hobbyist beekeepers. The monitoring levels were lowest in 2023 at 73.1% and highest in 2024 at 81.4%.

## 3. Results

### 3.1. Full Sample

**Figure 1:**
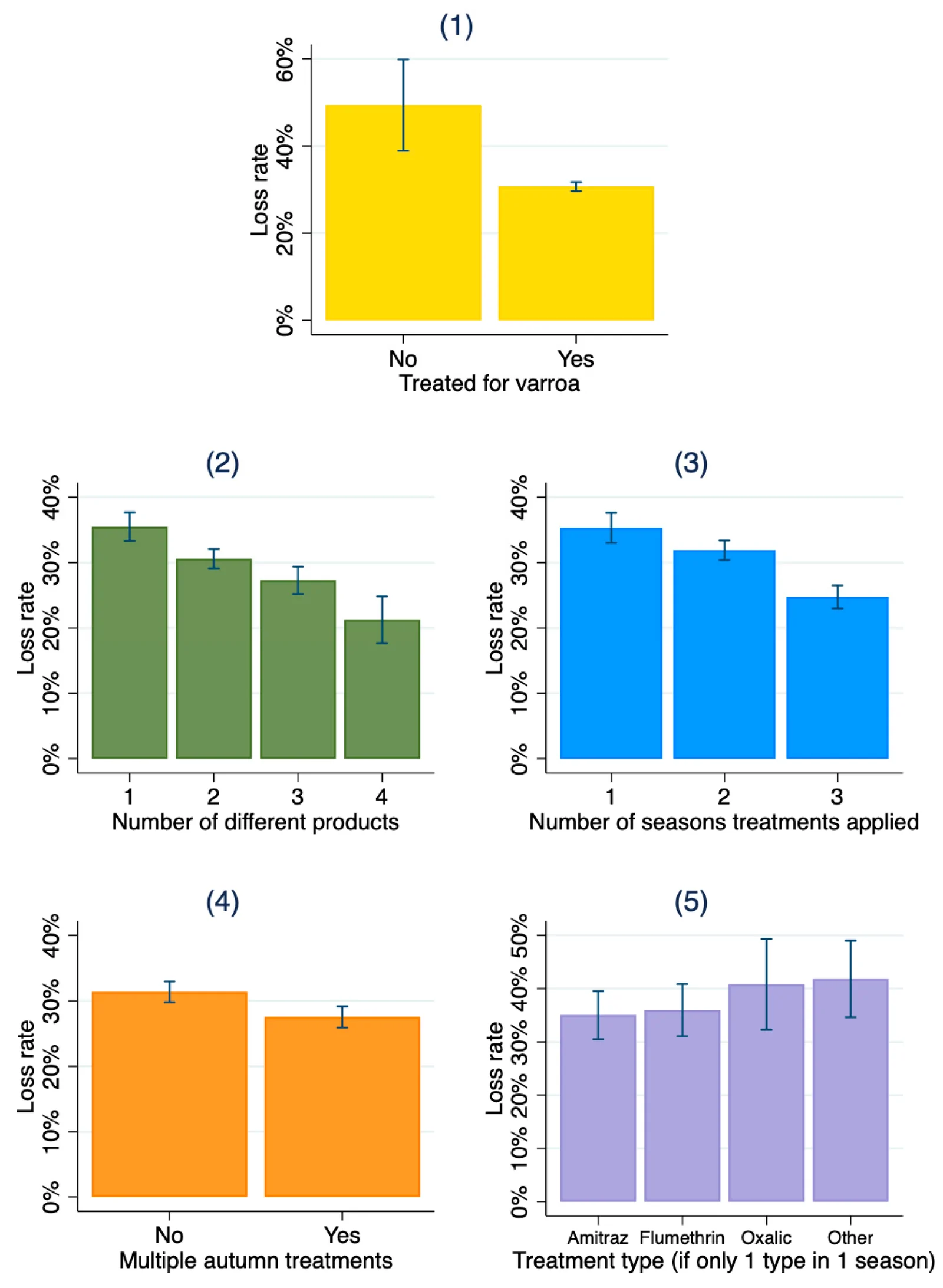
Loss rates among the full sample of hobbyist beekeepers.

Estimates from Equation (3) using the full sample of 5203 hobbyist beekeepers are presented in Figure 1 and Table 5. Year dummies, region dummies, and year × region interactions are included in the model but are not presented in the table for the sake of parsimony (Ref. [24] provides a detailed breakdown of winter losses by region and year). Heteroskedasticity-robust standard errors were calculated and are reported in the table.

Controlling for year, region, experience, and apiary size, hobbyist beekeepers who did not treat for varroa lost 49.4% of their colonies each winter (Figure 1, panel 1). Hobbyist beekeepers who treated for varroa lost 30.7% of their colonies over winter, a difference that is statistically significant at the 1% level.

Conditional on treating varroa, beekeepers who used one product lost 35.5% of their colonies over winter (panel 2). Beekeepers who used two different products lost 30.6% of their colonies. Beekeepers who used three different products lost 27.3% of their colonies. Beekeepers who used four or more products lost 21.2% of their colonies. All these differences are statistically significant at the 1% level.

Conditional on treating varroa, beekeepers who treated in only one season during the year lost 35.3% of their colonies over winter (panel 3). Beekeepers who treated in two seasons lost 31.9% of their colonies. Beekeepers who treated in three or four seasons lost 24.8% of their colonies. These differences are statistically significant at the 1% level.

Conditional on treating varroa in autumn, beekeepers who treated once in autumn lost 31.4% of their colonies over winter (panel 4). Beekeepers who treated multiple times in autumn lost 27.5% of their colonies over winter, a difference that is statistically significant at the 1% level.

In panel 5, the sample is restricted to 826 hobbyist beekeepers who treated with a single product in a single season during the year. Controlling for experience, apiary size, year, region, and their interaction, the effectiveness of amitraz, flumethrin, oxalic acid, and other treatments such as formic acid and thymol was statistically indistinguishable.

When controlling for apiary size, year, region, and their interaction, beekeeper experience does not have a statistically significant effect on expected loss rates (Table 5). However, when controlling for experience, year, region, and their interaction, apiary size is negatively associated with loss rates, a relationship that is statistically significant at the 1–10% levels across specifications.

### 3.2. Restricted Sample

**Figure 2:**
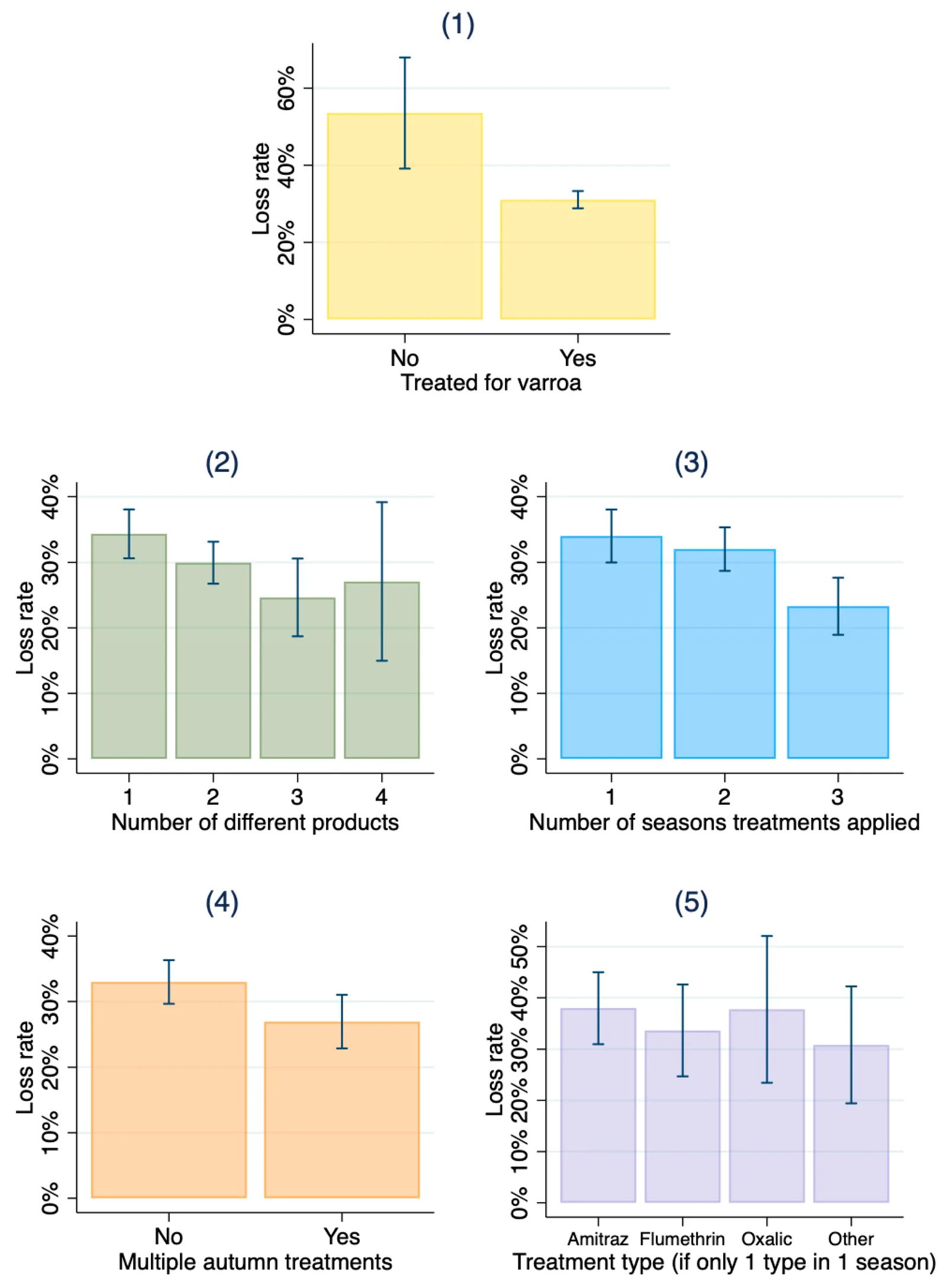
Loss rates among the restricted sample of hobbyist beekeepers who did not monitor varroa.

As noted above, our point estimates may be biased if beekeepers treat after finding extremely high mite loads during monitoring. Therefore, we re-estimated Equation (3) while restricting the sample to 1150 hobbyist beekeepers who did not monitor varroa. The results are presented in Table 6. As above, we include (but do not report) year dummies, region dummies, and year × region interactions, and all reported standard errors are heteroskedasticity-robust. Expected loss rates among beekeepers who did not monitor are also presented visually in Figure 2.

Among hobbyist beekeepers who did not monitor varroa, those who did not treat for varroa lost 53.5% of their colonies over winter (Figure 2, panel 1). Beekeepers who treated for varroa lost 31.1% of their colonies. This difference is statistically significant at the 1% level.

Conditional on treating varroa, beekeepers who used one product lost 34.4% of their colonies over winter (panel 2). Beekeepers who used two different products lost 30.0% of their colonies, a difference that is statistically significant at the 10% level. Beekeepers who used three different products lost 24.7% of their colonies, a difference that is statistically significant at the 1% level.

Conditional on treating varroa, there is no statistically discernible difference in loss rates between treating in one season of the year or two (panel 3). However, losses of beekeepers who treated in three or four seasons were 11.1 percentage points lower than for those who treated in only one season, significant at the 1% level.

Conditional on treating varroa in autumn, hobbyist beekeepers who treated once in autumn lost 33.0% of their colonies over winter (panel 4). Hobbyist beekeepers who treated multiple times in autumn lost 27.0% of their colonies over winter, a difference that is statistically significant at the 5% level.

As above, panel 5 restricts the sample to 292 hobbyist beekeepers who treated with a single product in a single season. Within this very restricted sample, no treatment was statistically more effective at reducing losses than any other.

## 4. Discussion

New Zealand beekeepers now attribute more over-winter losses to varroa and related complications than to all other reasons combined [24]. As such, effective varroa management is a major determinant of winter colony survival. In addition, New Zealand hobbyist beekeepers experience substantially higher over-winter losses than commercial beekeepers. For these reasons, we sought to quantify the benefits of varroa control for New Zealand hobbyist beekeepers. Here, we not only review our results, but we also provide additional perspective and practical advice that may be of use to hobbyist beekeepers.

### 4.1. Treating Varroa Increases Over-Winter Survival Rates

Our study reveals a stark difference between hobbyist beekeepers who treated varroa and those who did not. Using three years of survey data with over 5000 observations, we show that hobbyist beekeepers who did not treat for varroa lost 49.4% of their colonies over winter compared to a loss rate of 30.7% among hobbyist beekeepers who applied varroa treatment—a 37.9% reduction in loss rates. This is clear, compelling evidence that varroa management is important for colony survival. Even so, we regard this result with some caution because 98.7% of hobbyist beekeepers in our sample had engaged in some form of varroa control.

Despite evidence such as that presented in our study, treatment-free beekeeping is an active area of scientific research in Europe [46], with some studies reporting that beekeepers can avoid varroa treatment without experiencing colony losses. The famous example of Gotland Island is often cited as evidence of successful varroa treatment-free beekeeping [47]. We note that Gotland Island is a closed apiculture system with low hive density, in contrast to New Zealand’s open apiculture system with high hive density. In systems with high varroa pressure, inadequate intervention has been associated with poorer colony outcomes [48].

### 4.2. Using Multiple Types of Treatment Increases Over-Winter Survival Rates

Varroa treatments have different modes of action, so combining treatments can help prevent miticide resistance [49,50,51]. Among hobbyist beekeepers who treated for varroa, the most common number of treatment types used was two. Beekeepers who used a single product lost 35.5% of colonies compared to 30.6% among beekeepers who used two products, a reduction of 13.8%. Further reductions in loss rates occurred for hobbyists who used three or more products.

### 4.3. Treating in Multiple Seasons Increases Over-Winter Survival Rates

The seasonality of bees, varroa, and environmental conditions means no single intervention is sufficient. For example, it is already understood that treatment effectiveness varies by season and that timing varroa treatment appropriately is important for controlling mite levels [42,52,53].

Among hobbyist beekeepers who treated for varroa, most applied treatments in two different seasons during the year. Yet, loss rates were substantially lower when treatments extended into a third season. For example, beekeepers who treated in a single season lost 35.3% of colonies, compared to 24.8% among beekeepers who treated in three or more seasons, a reduction of 29.7%.

### 4.4. Applying Multiple Autumn Treatments Increases Over-Winter Survival Rates

Autumn is marked by the convergence of high varroa populations, the production of winter bees, and reinvasion, making colony outcomes in winter highly dependent on management during autumn [14,15]. Indeed, refs. [13,54] contend that autumn is the most critical time for varroa management.

Varroa populations increase throughout summer, so colonies may enter autumn with high but not visible mite loads [55]. That is, colonies may appear strong while still suffering significant mite burden, and colonies going into winter with high mite burdens are more likely to succumb, even if earlier interventions appeared to be adequate [15]. As such, multiple autumn treatments may influence over-winter survival rates.

Based on our results, hobbyist beekeepers who treated varroa a single time in autumn lost 31.4% of their colonies over winter compared to 27.5% for hobbyist beekeepers who treated more than once. That is, a second varroa treatment in autumn yielded a 12.4% reduction in expected over-winter loss rates. However, hobbyists need to keep in mind that resistance may eventuate unless products used to treat varroa are alternated.

### 4.5. No Single Varroa Treatment Type Outperformed Any Other

The relative effectiveness of different products for treating varroa receives considerable attention in discussions at beekeeping clubs. Resistance to miticides is also the subject of considerable attention in the scientific literature, including in New Zealand (e.g., [56]). Our results show that no single treatment type outperformed any other. We acknowledge that by limiting our sample to hobbyist beekeepers who used a single product in a single season (as a means of ensuring statistical identification), our approach is quite restrictive; that is, we do not consider single products applied in multiple seasons, multiple products applied in a single season, or combinations of products applied in different seasons. Moreover, while our survey data records the type of treatment used, it does not capture the timing of application or how treatments were applied; as such, our results may differ from those obtained in controlled laboratory environments.

### 4.6. Beekeeper Experience Has a Negligible Effect on Over-Winter Survival Rates

The over-winter loss rate among hobbyist beekeepers with more than five years of experience was statistically indistinguishable from the loss rate among hobbyist beekeepers with five or fewer years of experience. That is, beekeeper experience matters far less than whether varroa is treated, the number of different products used to treat varroa, the number of seasons in the year in which varroa treatment is applied, or whether varroa is treated multiple times in autumn. These results are consistent with broader empirical studies showing only weak associations between experience and performance [57,58].

The weak effect of experience suggests that mistakes persist regardless of time spent beekeeping, indicating that over-winter colony losses are not a beginner problem. Indeed, although the proportion of respondents with more than five years’ experience has increased over time (39.7% to 55.7%), colony loss rates have also risen during this time frame.

As colony conditions and management needs change over the years, practices may not be applied consistently, even among experienced beekeepers, because performance plateaus once a basic level of routine practice is established [59]. Many hobbyists may therefore appropriately be described as ‘permanent novices’, with perceived confidence increasing faster than demonstrated performance.

### 4.7. Varroa Monitoring Helps Beekeepers Optimise the Timing of Varroa Treatment

Finally, although not a focus of this analysis, we note that monitoring enhances the effectiveness of varroa treatment by facilitating optimised treatment timing [39]. Specifically, monitoring allows beekeepers to detect when mite infestations approach established treatment thresholds, enabling intervention before populations escalate. This is particularly critical in late summer and autumn, when mite populations and virus loads increase, and some treatments, such as organic products, may be insufficient in efficacy or duration to prevent over-winter colony loss [42].

Between 2023 and 2025, 22.3% of the hobbyist beekeepers who completed the NZCLS undertook no formal monitoring, consistent with many hobbyists avoiding monitoring due to a reluctance to kill bees, the perceived complexity of the methods, and/or a lack of confidence [60]. However, if hobbyist beekeepers only treat after seeing phoretic mites on adult bees (which generally indicates severe infestation), then intervention may be applied after it is too late for treatment to make a difference [61]. Ongoing training opportunities may be especially important in helping hobbyist beekeepers fine-tune their monitoring and treatment regimes [62].

### 4.8. Limitations

While the quantitative evidence supporting good varroa management is very strong, we note several caveats. First, our analysis is restricted to New Zealand hobbyist beekeepers. While we believe that the general insights apply equally to commercial beekeepers and to beekeepers operating in other countries, this hypothesis requires confirmation. Second, our analysis is based on survey data. As such, we acknowledge the possibility that non-respondents may systematically differ from respondents, leading to response bias. If, for example, non-respondents used fewer treatments and experienced lower losses than respondents, then the magnitudes of our estimates will be overstated. We view this as being an unlikely scenario, given that between 29% and 43% of all New Zealand beekeepers completed the survey during each of our study years (higher than the response rates obtained in any other COLOSS member country by a wide margin), but we cannot rule out this possibility. Third, we acknowledge other potential biases, including measurement error and omitted variable biases. Measurement error may occur if beekeepers do not accurately report their losses or their varroa management; while we believe the data accuracy to be high, further investigation may be needed to confirm this. Omitted variable bias may arise because the survey data does not record environmental conditions or forage availability, for example. Finally, while we have selected New Zealand hobbyist beekeepers as our focus for this analysis, we have not specifically analysed why hobbyists experienced higher losses than commercial operators. Complexity [60], monitoring [61], and education [62] are all explanations explored in the literature.

## 5. Conclusions

Varroa has been present in New Zealand since 2000 and is considered the biggest threat to managed honey bees worldwide. Although hobbyist beekeepers are aware of the threats posed by varroa, our study shows that simple awareness of varroa amongst hobbyist beekeepers is not enough to protect their colonies from winter mortality.

Even though varroa treatment use is extremely high among New Zealand hobbyist beekeepers, we show that substantial reductions in over-winter loss rates can be achieved through relatively modest changes in beekeeper behaviour. Using data from more than 5200 hobbyist beekeepers in all 16 regions over three years, we find that (in order of importance):

- When no varroa treatment is used, hobbyist beekeepers experienced high colony loss rates. Treating for varroa reduced loss rates by 20 percentage points.
- Using multiple products to treat for varroa further reduced loss rates. This is consistent with best practice to avoid chemical resistance.
- Hobbyist beekeepers who applied varroa treatments across multiple seasons in the year experienced lower loss rates than those who treated in only one season. Multiple treatments interrupted the mites’ reproductive cycle.
- Hobbyist beekeepers who treated for varroa multiple times in autumn experienced lower losses than those who treated only once in autumn. Again, this practice disrupted the mites’ reproductive cycle, lowering mite populations going into winter. However, applying the same product multiple times in succession (without altering products) may lead to resistance.
- Among beekeepers who used one treatment type in a single season, the choice of product made no statistically significant difference to colony survival.
- More experienced beekeepers experienced slightly lower loss rates, but the difference is negligible compared to treating for varroa, alternating products, and treating throughout the year. That is, experience does not prevent losses if the same mistakes are made year after year.

Although not a focus of this study, we also note that monitoring varroa infestation levels can help beekeepers optimise the timing of treatment, ensuring that treatments are most likely to be effective.

Bee clubs and respected local apiculture educators are key to spreading best practice—delivering clear, simple messages, followed by ongoing guidance reflecting the complexity and variability of effective varroa control. Addressing the underlying drivers behind reluctance to treat effectively could help with adoption of the key findings of this study.

## Figures and Tables

**Table 1:** Average over-winter loss rate across beekeeper size classes.

Year	Hobbyist (1–49 Colonies)	Sideliner (50–499 Colonies)	Commercial (500+ Colonies)
2025	33.4%	13.2%	13.0%
2024	33.3%	8.3%	9.6%
2023	24.0%	18.9%	12.2%
2022	32.1%	19.9%	13.4%
2021	28.6%	21.2%	14.7%
2020	26.9%	14.6%	10.8%
2019	22.9%	11.8%	9.9%

Source: [24].

**Table 2:** Number and percent of beekeepers who responded to the survey. Number and percent of colonies covered by the survey.

Year	No. of Respondents	% of All Registered Beekeepers with Bees	No. of Colonies Reported	% of All Registered Colonies
2025	1948	29.08%	148,860	30.04%
2024	2828	33.78%	153,856	28.80%
2023	3457	42.60%	208,044	35.00%

Source: [24].

**Table 3:** Common varroa controls in New Zealand.

Chemical Class	Active Ingredient	Mechanism	Product Name	NZ Registration
Amidine	Amitraz	Strips	Apitraz^®^	2018
Amidine	Amitraz	Strips	Apivar^®^	2003
Pyrethroid	Tau-fluvalinate	Strips	Apistan^®^	2000
Pyrethroid	Flumethrin	Strips	Bayvarol^®^	2000
Organic acid	Formic acid	Pads	Formic Pro^TM^	2017
Organic acid	Formic acid	Wick, Tray	(generic)	N/A
Organic acid	Oxalic acid	Strips	VarroxSan^®^	2025
Organic acid	Oxalic acid	Strips, Vaporisation, Dribbling	(generic)	N/A
Essential oil	Thymol	Tray	Apiguard^®^	2004
Essential oil	Thymol, Eucalyptol, Menthol, Camphor	Strips	Apilife VAR^®^	2005
Essential oil	Thymol	Wafers	Thymovar^®^	2006
Essential oil	Thymol	Strips, Wafers, Trays, Crystals	(generic)	N/A
Mineral oil	Mineral oil	Vaporisation	(generic)	N/A

Source: [33].

**Table 4:** Summary statistics.

	Year			
	2023	2024	2025	Total
*N*	2053	1907	1243	5203
Average loss rate as a percent (total *n* = 5203, standard deviation in parentheses)	26.3 (37.8)	33.8 (38.3)	34.2 (38.3)	30.9 (38.3)
Treated for varroa as a 1/0 variable (total *n* = 5203, percent in parentheses)				
No	40 (1.9%)	15 (0.8%)	15 (1.2%)	70 (1.3%)
Yes	2013 (98.1%)	1892 (99.2%)	1228 (98.8%)	5133 (98.7%)
Number of different products as a count variable, conditional on treating (total *n* = 5126, percent in parentheses)				
1	569 (28.3%)	450 (23.8%)	308 (25.1%)	1327 (25.9%)
2	954 (47.4%)	930 (49.3%)	518 (42.3%)	2402 (46.9%)
3	402 (20.0%)	405 (21.5%)	304 (24.8%)	1111 (21.7%)
4+	88 (4.4%)	102 (5.4%)	96 (7.8%)	286 (5.6%)
Number of seasons in which treatments applied as a count variable, conditional on treating (total *n* = 5104, percent in parentheses)				
1	532 (26.4%)	405 (21.5%)	250 (20.8%)	1187 (23.3%)
2	988 (49.1%)	948 (50.2%)	490 (40.7%)	2426 (47.5%)
3+	493 (24.5%)	534 (28.3%)	464 (38.6%)	1491 (29.2%)
Multiple autumn treatments as a 1/0 variable, conditional on treating in autumn (total *n* = 4137, percent in parentheses)				
No	874 (53.9%)	757 (50.9%)	516 (50.2%)	2147 (51.9%)
Yes	748 (46.1%)	730 (49.1%)	512 (49.8%)	1990 (48.1%)
Varroa treatment as a 1/0 variable, conditional on using only 1 treatment in 1 season (total *n* = 826, percent in parentheses)				
Amitraz	161 (41.4%)	105 (39.5%)	56 (32.7%)	322 (39.0%)
Flumethrin	151 (38.8%)	74 (27.8%)	47 (27.5%)	272 (32.9%)
Oxalic acid	28 (7.2%)	29 (10.9%)	34 (19.9%)	91 (11.0%)
Other	49 (12.6%)	58 (21.8%)	34 (19.9%)	141 (17.1%)
More than 5 years of experience as a 1/0 variable (total *n* = 5203, percent in parentheses)				
No	1237 (60.3%)	977 (51.2%)	551 (44.3%)	2765 (53.1%)
Yes	816 (39.7%)	930 (48.8%)	692 (55.7%)	2438 (46.9%)
Average apiary size as a continuous variable (standard deviation in parentheses)	3.29 (4.33)	4.12 (5.48)	4.19 (5.49)	3.81 (5.07)
Region as a 1/0 variable (total *n* = 5203, percent in parentheses)				
Upper North Island (Northland, Auckland, Coromandel)	477 (23.2%)	415 (21.8%)	284 (22.8%)	1176 (22.6%)
Middle North Island (Waikato, Bay of Plenty, Gisborne, Hawke’s Bay)	376 (18.3%)	374 (19.6%)	263 (21.2%)	1013 (19.5%)
Lower North Island (Taranaki, Manawatū-Wanganui, Wairarapa, Wellington)	439 (21.4%)	400 (21.0%)	255 (20.5%)	1094 (21.0%)
Upper South Island (Marlborough, Tasman, Nelson)	138 (6.7%)	126 (6.6%)	90 (7.2%)	354 (6.8%)
Middle South Island (Canterbury, West Coast)	327 (15.9%)	321 (16.8%)	180 (14.5%)	828 (15.9%)
Lower South Island (Otago, Southland)	296 (14.4%)	271 (14.2%)	171 (13.8%)	738 (14.2%)
Monitored for varroa as a 1/0 variable (total *n* = 5167, percent in parentheses)				
No	542 (26.9%)	354 (18.6%)	254 (20.4%)	1150 (22.3%)
Yes	1475 (73.1%)	1553 (81.4%)	989 (79.6%)	4017 (77.7%)

**Table 5:** Winter colony loss rates among hobbyist beekeepers, 2023–2025 (marginal effects reported).

	(1)		(2)		(3)		(4)		(5)	
Treated for varroa	−0.172	***								
	(0.047)									
2 different products			−0.047	***						
			(0.013)							
3 different products			−0.081	***						
			(0.015)							
4+ different products			−0.148	***						
			(0.024)							
Treated in 2 seasons					−0.033	**				
					(0.013)					
Treated in 3+ seasons					−0.106	***				
					(0.015)					
Multiple autumn treatments							−0.038	***		
							(0.012)			
Varroa treatment (if only 1 treatment)										
Amitraz									0.010	
									(0.034)	
Flumethrin									0.058	
									(0.049)	
Oxalic acid									0.068	
									(0.044)	
More than 5 years of experience	−0.005		−0.006		−0.004		−0.003		−0.048	
	(0.011)		(0.011)		(0.011)		(0.012)		(0.030)	
Log number of colonies	−0.040	***	−0.032	***	−0.032	***	−0.035	***	−0.032	*
	(0.006)		(0.006)		(0.006)		(0.007)		(0.018)	
Year dummies	Yes		Yes		Yes		Yes		Yes	
Region dummies	Yes		Yes		Yes		Yes		Yes	
Year × Region interactions	Yes		Yes		Yes		Yes		Yes	
Number of observations	5203		5126		5104		4137		826	
Pseudo R-squared	0.016		0.019		0.020		0.014		0.027	
AIC	6376.06		6240.58		6204.25		4976.43		1106.41	
BIC	6513.76		6391.05		6348.08		5109.31		1214.89	

*** *p* < 0.01, ** *p* < 0.05, * *p* < 0.10. Heteroskedasticity-robust standard errors are shown in parentheses.

**Table 6:** Winter colony loss rates among hobbyist beekeepers who undertook no monitoring, 2023–2025 (marginal effects reported).

	(1)		(2)		(3)		(4)		(5)	
Treated for varroa	−0.207	***								
	(0.066)									
2 different products			−0.043	*						
			(0.025)							
3 different products			−0.099	***						
			(0.038)							
4+ different products			−0.073							
			(0.068)							
Treated in 2 seasons					−0.019					
					(0.026)					
Treated in 3+ seasons					−0.111	***				
					(0.032)					
Multiple autumn treatments							−0.061	**		
							(0.028)			
Varroa treatment (if only 1 treatment)										
Amitraz									−0.043	
									(0.059)	
Flumethrin									−0.002	
									(0.081)	
Oxalic acid									−0.072	
									(0.069)	
More than 5 years of experience	−0.025		−0.024		−0.022		−0.028		−0.052	
	(0.023)		(0.023)		(0.023)		(0.027)		(0.049)	
Log number of colonies	−0.037	***	−0.030	**	−0.032	**	−0.034	**	−0.004	
	(0.014)		(0.014)		(0.014)		(0.016)		(0.029)	
Year dummies	Yes		Yes		Yes		Yes		Yes	
Region dummies	Yes		Yes		Yes		Yes		Yes	
Year × Region interactions	Yes		Yes		Yes		Yes		Yes	
Number of observations	1150		1113		1114		832		292	
Adjusted R-squared	0.021		0.018		0.021		0.017		0.049	
AIC	1449.55		1398.43		1386.94		1051.33		407.66	
BIC	1555.55		1514.21		1497.15		1150.54		492.23	

*** *p* < 0.01, ** *p* < 0.05, * *p* < 0.10. Heteroskedasticity-robust standard errors are shown in parentheses.

## Data Availability

Results from the New Zealand Colony Loss Survey form the basis of this research. Most of these data are available from the New Zealand Ministry for Primary Industries (https://www.mpi.govt.nz/biosecurity/how-to-find-report-and-prevent-pests-and-diseases/bee-biosecurity/bee-colony-loss-survey, accessed on 1 July 2026). Additional data may be made upon reasonable written request to the corresponding author.

## Abbreviations

The following abbreviations are used in this manuscript:

COLOSS	Prevention of Honey Bee Colony Losses
DWV	Deformed wing virus
NZCLS	New Zealand Colony Loss Survey
